# Metabolomic Profiling in Individuals with a Failing Kidney Allograft

**DOI:** 10.1371/journal.pone.0169077

**Published:** 2017-01-04

**Authors:** Roberto Bassi, Monika A. Niewczas, Luigi Biancone, Stefania Bussolino, Sai Merugumala, Sara Tezza, Francesca D’Addio, Moufida Ben Nasr, Alessandro Valderrama-Vasquez, Vera Usuelli, Valentina De Zan, Basset El Essawy, Massimo Venturini, Antonio Secchi, Francesco De Cobelli, Alexander Lin, Anil Chandraker, Paolo Fiorina

**Affiliations:** 1 Nephrology Division, Boston Children’s Hospital, Harvard Medical School, Boston, MA, United States of America; 2 Transplant Medicine, IRCCS Ospedale San Raffaele, Milan, Italy; 3 Section on Genetics and Epidemiology, Joslin Diabetes Center, Harvard Medical School, Boston, MA, United States of America; 4 San Giovanni Battista Hospital and University of Turin, Division of Nephrology, Dialysis, and Transplantation, Turin, Italy; 5 Biomedical Engineering, University of Texas, Austin, TX, United States of America; 6 Universita’ Vita-Salute San Raffaele, Milan, Italy; 7 Medicine, Al-Azhar University, Cairo, Egypt; 8 Radiology, San Raffaele Scientific Institute, Milan, Italy; 9 Center for Clinical Spectroscopy, Department of Radiology, Brigham and Women's Hospital, Harvard Medical School, Boston, MA, United States of America; 10 Transplantation Research Center, Brigham and Women’s Hospital, Harvard Medical School, Boston, MA, United States of America; Universidade Federal de Sao Paulo, BRAZIL

## Abstract

**Background:**

Alteration of certain metabolites may play a role in the pathophysiology of renal allograft disease.

**Methods:**

To explore metabolomic abnormalities in individuals with a failing kidney allograft, we analyzed by liquid chromatography-mass spectrometry (LC-MS/MS; for *ex vivo* profiling of serum and urine) and two dimensional correlated spectroscopy (2D COSY; for *in vivo* study of the kidney graft) 40 subjects with varying degrees of chronic allograft dysfunction stratified by tertiles of glomerular filtration rate (GFR; T1, T2, T3). Ten healthy non-allograft individuals were chosen as controls.

**Results:**

LC-MS/MS analysis revealed a dose-response association between GFR and serum concentration of tryptophan, glutamine, dimethylarginine isomers (asymmetric [A]DMA and symmetric [S]DMA) and short-chain acylcarnitines (C4 and C12), (test for trend: T1-T3 = p<0.05; p = 0.01; p<0.001; p = 0.01; p = 0.01; p<0.05, respectively). The same association was found between GFR and urinary levels of histidine, DOPA, dopamine, carnosine, SDMA and ADMA (test for trend: T1-T3 = p<0.05; p<0.01; p = 0.001; p<0.05; p = 0.001; p<0.001; p<0.01, respectively). *In vivo* 2D COSY of the kidney allograft revealed significant reduction in the parenchymal content of choline, creatine, taurine and threonine (all: p<0.05) in individuals with lower GFR levels.

**Conclusions:**

We report an association between renal function and altered metabolomic profile in renal transplant individuals with different degrees of kidney graft function.

## Introduction

Kidney transplantation has become the most widespread organ engrafting procedure [1]. While advances in immunosuppressive protocols have reduced the incidence of kidney acute rejection over the years [2], long-term outcome of the kidney allograft remains affected by the persistence of chronic allograft dysfunction [3–6]. The success of a renal transplant strictly depends on the ability of monitoring transplant recipients and responsively changing their medications. Unfortunately, we are still relying on the measurement of serum creatinine levels and proteinuria to assess kidney function, which are non-specific and insensitive markers [7,8,9] and whose increase may underlie an already predominantly lost kidney function [8, 9]. Also, metabolic tests and imaging techniques which are routinely employed to detect graft dysfunction, in some circumstances do not provide adequate specificity, sensitivity, or accuracy [7, 10]. Thus, follow-up biopsies, both inconvenient to the patient and associated with expensive histopathological analysis, are required to reach a definitive diagnosis [11]. The appearance of novel techniques that allow the detection of unprecedentedly discovered pathways or unidentified metabolites, may lead to a whole new era of patient management, particularly the use of novel "omics" may generate opportunities unexplored thus far, ideally bypassing the shortcomings of the current routine diagnostic tools. Metabolomics has the potential to perform an unbiased, non-targeted and dynamic analysis of low molecular mass cellular products, thus making it an ideal candidate for the discovery of new potential markers of renal graft function in the transplant patient [12,13,14,15]. Multiple studies report the association between certain immunosuppressive schemes and specific metabolic alterations in urine and serum of transplant patients [16–18] while others propose a relationship between acute renal allograft rejection and urine metabolic profile [19]. Metabolite alteration may also accompany the progression of chronic kidney allograft dysfunction and this may be relevant for the outcome both in terms of graft survival and health of the patient. Thus, aiming to explore the profile of metabolomic abnormalities induced by the progressive reduction of kidney function and their potential impact on kidney graft function, we took advantage of two complementary approaches: liquid chromatography-mass spectrometry (LC-MS/MS) for targeted metabolomic profiling of serum and urine [20] and two dimensional correlated spectroscopy (2D COSY) [21, 22] for the *in vivo* metabolomic profiling of the kidney allograft, in a population of individuals with different degrees of graft dysfunction, defined by progressively lower levels of glomerular filtration rate (GFR) and a pool of healthy non-allograft individuals controls. We thus performed an analysis of the transplant individual at the serum, urine and kidney graft level by taking advantage of the latest analytical techniques, in order to gain insights into the metabolomic abnormalities evident in individuals with failing kidney allografts.

## Materials and Methods

A complete description of methods is offered in the S1 Data.

### Patient characteristics

Forty kidney transplant individuals, with at least 6 months of follow-up after transplantation, were admitted for post-transplantation routine analysis. After clinical evaluation, individuals were enrolled in the study and assigned to different groups according to degree of allograft function impairment. Transplant patients were then stratified in tertiles according to GFR distribution (T1, T2 and T3) as shown in Table 1. Exclusion criteria were defined as (i) GFR < 25 ml/min; (ii) serum creatinine > 3.0 mg/dl; (iii) severe uncontrolled arterial hypertension; and (iv) arterial renal stenosis (assessed with Color Doppler Ultrasonography). Finally, the control group (Ctrl) consisted of ten healthy individuals with normal renal function. Data were obtained after individuals’ written consent. The study protocol was conducted after Institutional Review Board approval. A blinded code was assigned to each participating patient. Kidney transplant recipients did not differ with regard to donor age, HLA match, panel of reactive antibodies, cold ischemia time, rejection rate, cytomegalovirus infection and lymphoproliferative diseases across the various renal function strata.

**Table 1:** Demographic and metabolic characteristics of kidney transplant individuals. Results are expressed as median (25^th^, 75^th^ percentile).

	T1 (56–108 ml/min)	T2 (46–55 ml/min)	T3 (21–39 ml/min)	p-value
Age	56.0 (44.5, 62.0)	62.0 (53.0, 65.0)	55.0 (48.0, 65.0)	ns
Pre-transplant dialysis duration (months)	35.0 (15.5, 112.5)	53.0 (43.0, 90.0)	78.0 (15.7, 102.0)	ns
Follow-up (months)	75.0 (48.5, 115.0)	77.0 (30.0, 118.0)	64.5 (15.7, 185.8)	ns
Systolic blood pressure (mmHg)	130.0 (127.5, 150.0)	130.0 (130.0, 140.0)	140.0 (121.3, 152.5)	ns
Diastolic blood pressure (mmHg)	80.0 (75.0, 90.0)	80.0 (80.0, 85.0)	75.5 (70.0, 80.0)	ns
Cholesterol (mg/dl)	160.0 (147.5, 187.0)	180.0 (155.0, 213.0)	204.5 (176.8, 240.0)	ns
Triglycerides (mg/dl)	106.0 (72.5, 159.5)	166.0 (83.0, 209.0)	140.0 (100.8, 198.8)	ns
BUN (mg/dl)	56.5 (49.5, 76.5)	76.0 (67.0, 103.8)	104 (88.75, 150.8)	0.009
GFR (ml/min/1.73m^2^)	65.0 (60.0, 83.5)	50.0 (48.0, 55.0)	34.5 (24.2, 35.7)	*by design*
S-Creatinine (mg/dl)	1.3 (1.2, 1.6)	1.5 (1.5, 1.8)	2.4 (2.0, 2.7)	<0.0001
AER (g/day)	0.1 (0.1, 0.2)	0.3 (0.1, 0.4)	1.0 (0.2, 2.5)	0.009

**Abbreviations.** Male (M); female (F); blood urea nitrogen (BUN); glomerular filtration rate (GFR); albumin excretion rate (AER).

### Metabolomics protocol

To gain greater insight into the metabolome of the kidney transplant patient, we opted to use a novel and unique composite approach to define the of *ex vivo* (serum and urine) and *in vivo* metabolomic profile of kidney transplant individuals by using LC-MS/MS, FIA-MS/MS (n = 40 patients) and 2D COSY [22] with subsequent 3D-image transformation [23], performed on a subgroup (n = 15) of renal transplant individuals. For additional details on the metabolomics protocol, please refer to S1 Data. The Human Metabolome DataBase (HMDB, http://www.hmdb.ca/) was used to study the metabolic pathways at the base of the observed molecular alterations and to hypothesize potential effects of these on graft function.

### Statistical analysis

**Fig 1:**
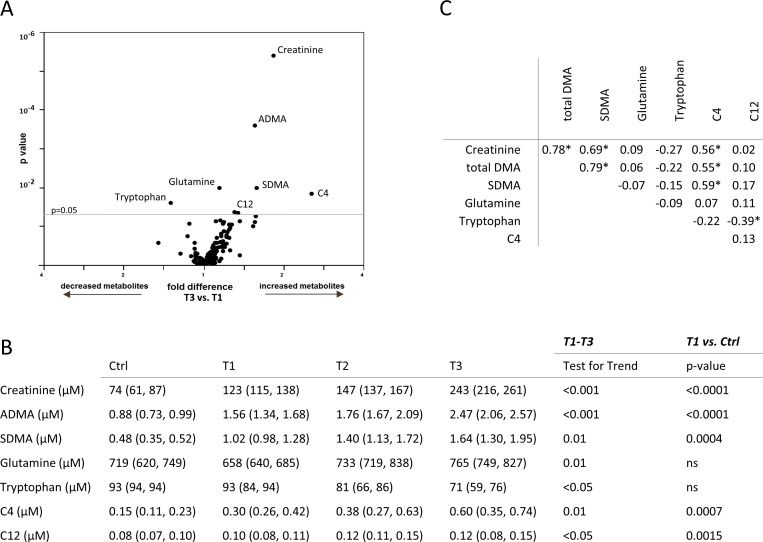
(A) Multivariate analysis (volcano plot) of common metabolites measured in the serum on the Biocrates platform and their association with glomerular filtration rate (GFR; T1-T3) are reported as fold difference (x-axis), and nominal significance is presented on the y-axis. (B) Serum metabolites significantly different among patients with varying renal function are shown in the kidney transplant recipient (T1, T2, T3) and the control (Ctrl) group. (C) Spearman nonparametric correlation matrix among the metabolites in serum significantly associated with varying kidney transplant function. Correlation coefficients are presented. Significant associations are marked with an asterisk (*).

**Fig 2:**
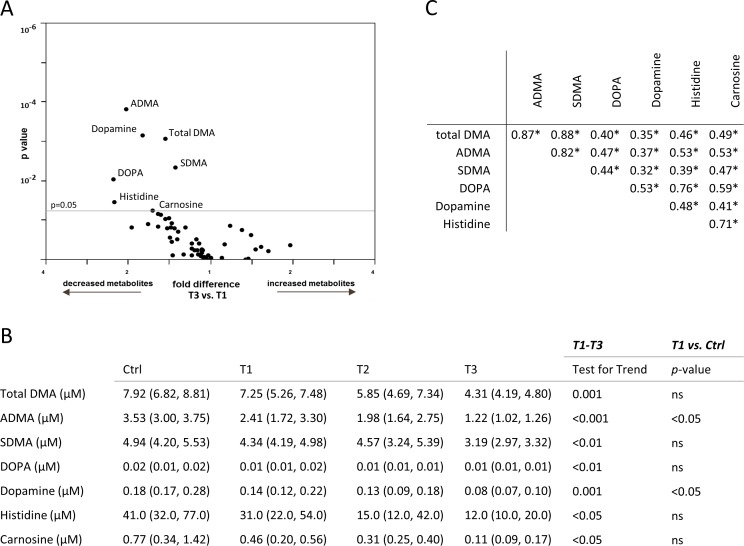
(A) Multivariate analysis (volcano plot) of common metabolites measured in the urine on the Biocrates platform and their association with glomerular filtration rate (GFR; T1-T3) are reported as fold difference (x-axis), and nominal significance is presented on the y-axis. (B) Urinary metabolites significantly different among patients with varying renal function are presented in the kidney transplant recipient (T1, T2, T3) and the control (Ctrl) group. (C) Spearman nonparametric correlation matrix among the metabolites in urine significantly associated with varying kidney transplant function. Correlation coefficients are presented. Significant associations are marked with an asterisk (*).

Serum markers were presented as median (25th, 75th percentiles). Urinary markers normalized to creatinine were presented as median (25th, 75th percentiles). Serum and urinary metabolites present in at least 80% of the study subjects were designated as common and subjected to further analysis. Kidney transplant recipients were stratified according to the distribution of renal function (T1, T2, T3), in which T3 represented subjects with impaired graft function, T2 subjects with fairly conserved renal allograft function and T1 represented subjects with well-preserved renal function, respectively. Multivariate analysis (volcano plot) of common metabolites represents a fold difference (x-axis) between mean values of the metabolites within T3 and T1 strata respectively, whereas nominal significance is presented on the y-axis (Figs 1 and 2). Differences among the groups were evaluated in the general linear model based on the metabolites transformed to their logarithms (base 10). Study groups were treated in a categorical (T3 vs. T1, T1 vs. Ctrl) or an ordinal way (T1-T3) appropriately. Spearman nonparametric correlation matrix was created among kidney transplant recipients to evaluate correlations among the metabolites. Correlation coefficients are presented. All tests were two-sided, and a p value of less than 0.05 was considered indicative of statistical significance. Data analysis was performed using SAS version 9.3 (SAS Institute, Cary, NC).

## Results

### Individual characteristics

Forty kidney transplant individuals were enrolled in our cross-sectional study and stratified according to tertiles of GFR distribution as follows: T1 = 56–108 ml/min; T2 = 46–55 ml/min; and T3 = 21–39 ml/min. Individuals among groups did not show major differences in terms of demographic characteristics, lipid profile or blood pressure measurements (Table 1), while mean group comparison revealed significant differences in blood urea nitrogen, serum creatinine and albumin excretion rate among T1, T2 and T3 (Table 1).

### Ex vivo LC-MS/MS and FIA-MS/MS in kidney transplant individuals with different degrees of graft function

We took advantage of the AbsoluteIDQ^TM^ p180 kit assay (BIOCRATES Life Sciences AG) to determine serum and urinary concentration of 190 metabolites divided as follows: amines (amino acids and biogenic amines), acylcarnitines, phosphatidylcolines, sphingomyelins, lysophosphatidylcolines and hexose. The majority of the biochemical classes of metabolites were commonly detected in serum except for acylcarnitines, for which the detectability was 37%. On the contrary, there were two major biochemical classes of easily detectable metabolites in urine: amino acid and biogenic amines (88%) and acylcarnitines (46%). Finally, all lipid metabolites were below the limit of method detection in the urine samples (S1 Table).

### Serum metabolomic profiling

Protein or amino acid metabolism alterations, dietary deficiencies, increased catabolic degradation and inflammation are some of the causes behind metabolite abnormalities in serum among kidney graft individuals [24]. In our cohort, glutamine was progressively higher in kidney transplant individuals with impaired GFR (T3) as compared to patients with more preserved kidney function (T1) (T3 = 765 [749, 827] vs. T1 = 658 [640, 685] μM, p = 0.01; Fig 1A and 1B). Conversely, serum tryptophan was reduced in patients with lower GFR (T3) as compared to T1 patients (T3 = 71 [59, 76] vs. T1 = 93 [84, 94] μM, p<0.05; Fig 1A and 1B). Low serum tryptophan concentrations have been linked to inflammation and regulation of the immune response; in particular, indoleamine 2,3-dioxygenase (IDO)-mediated tryptophan catabolism has been reported during allograft rejection [25].

Among biogenic amines, dimethylarginine (DMA) analogues showed significant differences among groups. Specifically, asymmetric (A)DMA was increased in patients with reduced GFR (T3 = 2.47 [2.06, 2.57] vs. T1 = 1.56 [1.34, 1.68] μM, p<0.001; Fig 1A and 1B). Comparison of ADMA between T1 patients and control individuals revealed increases in serum ADMA levels in kidney recipients but with preserved renal function (T1 vs. Ctrl = 0.88 [0.73, 0.99] μM, p<0.0001; Fig 1B). Similarly, symmetric DMA (S)DMA was increased in patients with low GFR (T3 = 1.64 [1.30, 1.95] vs. T1 = 1.02 [0.98, 1.28] μM, p = 0.01; Fig 1A and 1B) and SDMA serum concentration variations were proportional to kidney graft performance (test for trend [T1-T3]: p = 0.01; Fig 1 B). Reference individuals with normal renal function displayed lower SDMA levels as compared to T1 patients (T1 vs. Ctrl = 0.48 [0.35, 0.52] μM, p = 0.0004; Fig 1B). Methylarginine isomers have been previously reported to be altered in individuals with chronic renal failure [26], perpetrating kidney damage through inhibition of nitric oxide synthase activity, induction of collagen and TGF-β1 synthesis and constituting independent causes of mortality and cardiovascular risk [27].

A common finding during renal insufficiency is the elevation of acylcarnitine serum content, most likely due to defective kidney excretion [28]. In the sample of patients under study, butyrylcarnitine (C4) and dodecanoylcarnitine (C12) were significantly higher in patients with worse graft function (C4: T3 = 0.60 [0.35, 0.74] vs. T1 = 0.30 [0.26, 0.42] μM, p = 0.01; C12: T3 = 0.12 [0.08, 0.15] vs. T1 = 0.10 [0.08, 0.11] μM, p<0.05; Fig 1A and 1B). Both C4 and C12 were significantly different between control individuals and kidney graft patients with conserved renal function (C4: T1 vs. Ctrl = 0.15 [0.11, 0.23] μM, p = 0.0007; C12: T1 vs. Ctrl = 0.08 [0.07, 0.10] μM, p = 0.0015; Fig 1B). These alterations are also consistent with the impaired fatty acid metabolism and subsequent acylcarnitine accumulation that occur during renal failure.

Spearman correlation matrix of serum metabolites significantly associated with kidney graft function revealed that only certain metabolites were correlated with each other. Total DMA, SDMA and acylcarntine C4 were significantly correlated. Interestingly, there was also an inverse association between acylcarnitine C12 and tryptophan. Glutamine did not correlate with any other metabolite in the studied matrix (Fig 1C).

### Urine metabolomic profiling

Urinary levels of amino acids and biogenic amines were overall reduced in individuals with poor graft function, pointing to reduced biosynthesis, enhanced catabolism or poor filtration of these classes of metabolites. Urinary histidine was reduced in T3 kidney graft patients as compared to patients with more conserved graft function (T3 = 12.0 [10.0, 20.0] vs. T1 = 31.0 [22.0, 54.0] μM, p<0.05; Fig 2A and 2B). Histidine is an anti-inflammatory and anti-oxidant factor, and its decrease has been associated with systemic inflammation and increased mortality in individuals with poor kidney function [29].

Among biogenic amines, the urinary concentration of carnosine was reduced in patients with a failing graft (T3 = 0.11 [0.09, 0.17] vs. T1 = 0.46 [0.20, 0.56] μM, p<0.05; Fig 2A and 2B). Similarly, free urinary dopamine was decreased in T3 individuals compared to T1 (T3 = 0.08 [0.07, 0.10] vs. T1 = 0.14 [0.12, 0.22] μM, p<0.001) and Ctrl individuals (T1 vs. Ctrl = 0.18 [0.17, 0.28] μM, p<0.05; Fig 2A and 2B). Urinary DOPA (a metabolic precursor of dopamine), followed the same pattern as dopamine (Fig 2A and 2B), and finally, total DMA and its two analogues ADMA and SDMA were lower in patients in the T3 group (total DMA: T3 = 4.31 [4.19, 4.80] vs. T1 = 7.25 [5.26, 7.48] μM, p = 0.001; ADMA: T3 = 1.22 [1.02, 1.26] vs. T1 = 2.41 [1.72, 3.30] μM, p<0.001; SDMA: T3 = 3.19 [2.97, 3.32] vs. T1 = 4.34 [4.19, 4.98] μM, p<0.01; Fig 2A and 2B). Notably, reduction in urinary ADMA, and in general disturbance of nitric oxide metabolism, have been recently associated with renal graft failure and increased mortality in individuals following kidney transplantation [30].

Spearman correlation matrix of urinary metabolites significantly associated with kidney graft function revealed that all the respective metabolites were significantly correlated with each other (Fig 2C).

### In vivo 2D COSY spectroscopy

**Fig 3:**
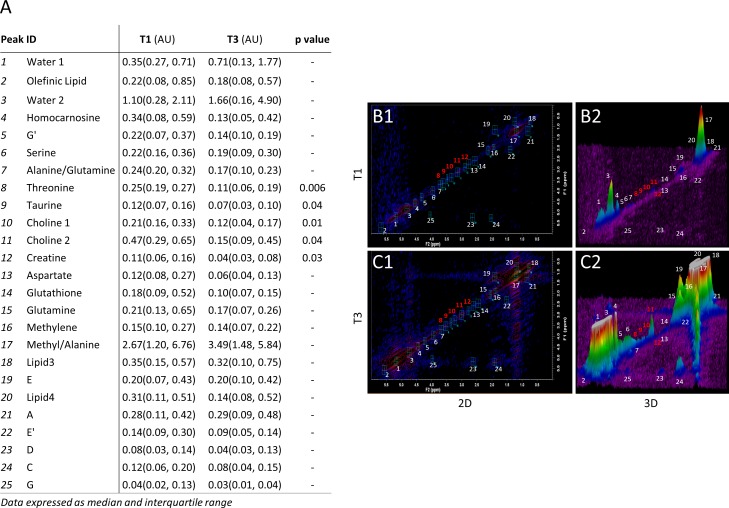
Two dimensional Correlated Spectroscopy (2D COSY) results of the kidney allograft. (A) Table of 2D COSYcrosspeak volumes shows significantly lower threonine, taurine, creatine and choline content in T3 individuals with low glomerular filtration rate and severe allograft dysfunction when compared to T1 individuals with more conserved graft function. “-”indicates a p value greater than 0.05. (B) Representative 2D COSY spectra show higher content of lipid-derived metabolites and reduced levels of threonine, taurine, creatine and choline in T3 individuals carrying a failing allograft. B1 shows a topological map of crosspeaks and B2 shows the 3D reconstruction. (C) Representative 2D COSY of T1 allograft patients with more conserved graft function with two-dimensional (C1) and three-dimensional (C2) reconstruction of the 2D COSY data. Data are expressed as median (25^th^, 75^th^ percentile). **Abbreviations.** Arbitrary Units (AU).

A subgroup of fifteen individuals (n = 5 from each GFR tertile subgroup) underwent 2D COSY examination for *in vivo* analysis of the transplanted kidney (Fig 3B1–3C1). Subsequently, additional 3D image post-processing was performed to better visualize and compare the differences among resonance and crosspeaks composing the spectra obtained from T1 and T3 groups (Fig 3A and 3B2–3C2), and 25 metabolites were identified. Mean comparison of the crosspeak volumes revealed differences in the concentrations of the amino acids taurine, which acts as an antioxidant agent and prevents lipid peroxidation of mesangial and tubular epithelial cells [31], and threonine, whose role remains obscure in the context of renal function (Fig 3A, 3C1 and 3C2). Both amino acids were found to be significantly reduced in the T3 group as compared to the T1 (Taurine: T3 = 0.07 [0.03, 0.10] vs. T1 = 0.12 [0.07, 0.16] arbitrary units [AU], p = 0.04; Threonine: T3 = 0.11 [0.06, 0.19] vs. T1 = 0.25 [0.19, 0.27] AU, p = 0.006; Fig 3A, 3B1, 3B2, 3C1 and 3C2). Among other metabolites identified by *in vivo* 2D COSY examination, choline, an essential nutrient with a pivotal role in the synthesis of cell membranes and neurotransmitters [32] (e.g. acetylcholine), was significantly reduced in T3 individuals as compared to T1 (Choline 1: T3 = 0.12 [0.04, 0.17] vs. T1 = 0.21 [0.16, 0.33] AU, p = 0.01; Choline 2: T3 = 0.15 [0.09, 0.45] vs. T1 = 0.47 [0.29, 0.65] AU; p = 0.04, Fig 3A, 3B1, 3B2, 3C1 and 3C2). Similarly, creatine, whose major function is to transport high energy groups from their site of production (mitochondria) to the site of ATP consumption in the cytoplasm [33], was depleted in kidneys from T3 allograft individuals compared to T1 patients (T3 = 0.04 [0.03, 0.08] vs. T1 = 0.11 [0.06, 0.16] AU, p = 0.03, Fig 3A, 3B1, 3B2, 3C1 and 3C2). Taken together, these data suggest that in kidney transplant individuals, low GFR may be associated with reduced metabolism/high-energy levels and reduced cellularity of the renal graft.

## Discussion

In this work, we have characterized the metabolite profile of biofluids (i.e. serum, urine) and of kidney allograft parenchyma in transplanted individuals with varying degrees of filtration impairment, by using novel analytical techniques that allow unbiased quantification of the molecular alterations associated with chronic allograft dysfunction [34], with the goal of defining the association between kidney allograft dysfunction and metabolomic fingerprint. Modifications in specific metabolites have been shown to be involved, either as a cause or symptom, in kidney disease. For instance, circulating amines (i.e. amino acids and biogenic amines) are promptly altered during the early phases of kidney impairment [34], and the more the graft fails, the more the imbalance becomes clear. These alterations can usually be attributed to increased protein degradation, inflammation [24, 35] or protein malnutrition. Accordingly, we showed that serum tryptophan alterations began to appear in T1 kidney allograft patients, to worsen in poor allograft function patients (T3) with a dose response trend, and this decline was not explained by urinary losses. Low serum tryptophan can be explained by an accelerated breakdown rate by the immunomodulatory enzyme IDO, due to an excess of inflammation/immune activation [25]. Evidence of a parallel reduction in urinary tryptophan in T3 individuals, who had poor allograft function, points to systemic exhaustion of this amino acid, rather than localized waste. Conversely, high serum glutamine can be explained by the substantial reduction in glutamine uptake that often takes place during chronic renal disease [36], and this is further confirmed by the reduction in urinary glutamine in individuals with GFR impairment. However, apart from the evidence that glutamine catabolism is one of the major determinants of ammonemia in these patients, the kinetics of glutamine in renal dysfunction are still largely unknown [37]. Progressive increase in the concentration of serum DMA derivatives coupled with decrease in their urinary excretion was also evident in individuals with more severe graft dysfunction. Low GFR can explain reduced excretion and serum accumulation of ADMA and SDMA, also confirming their classification as toxic uremic retention solutes [38]. DMA isomers appear to induce kidney damage through inhibition of nitric oxide synthase, induction of the synthesis of collagen and TGF-β1 and sodium retention (22), supporting the hypothesis that there is a relationship between ADMA and hypertension or glomerulosclerosis, two main determinants of kidney injury progression [39]. Finally, higher serum concentration of short-chain acylcarnitines (C4 and C12) in T3 patients can be attributed to the loss of renal parenchyma typical of long-term renal failure that, by removing a source of endogenous carnitine synthesis (thus reducing the handling and consumption of acylcarnitines), impairs the ability of the kidney to excrete acylcarnitine into the urine [40]. Finally, decreased levels of the branched chain amino acids have been described in the presence of advanced chronic kidney disease in some reports [41]. In our study, serum levels of leucine, isoleucine and valine did not differ between control groups and kidney transplant recipients with varying renal function, most probably due to the overall good nutritional status across groups of subjects under study.

Interesting results are also evident from urine mass spectrometry analysis. DOPA and dopamine were reduced in T3 transplant individuals as compared to patients with more conserved GFR. In the kidney, dopamine, when coupled to D_1_-like receptors in the proximal tubule, causes inhibition of sodium reabsorption by blocking Na/H-exchanger and Na/K-ATPase activity, thus regulating blood pressure. Notably, the absence of the same findings in the serum points to a reduction in dopamine synthesis at the kidney level—evidence previously linked with onset of hypertension. Reduction in urinary ADMA has also been associated with reduction in the lifespan of the kidney graft and overall mortality in kidney transplant patients [30].

Changes in the *in vivo* NMR spectroscopy profile of certain metabolites often precede morphological or symptomatic changes in the kidney, brain, breast, and other organs [42–46]. Although traditional 1D-NMR spectroscopy is sufficient to observe distinct functional groups in small molecules, many overlapping resonances in complex molecules can render the interpretation of peaks more difficult [47]. The use of 2D COSY circumvents this challenge by introducing a second dimension to the spectrum derived from the graft [48], while additional 3D image transformation adds further spatial detail to the examination. In our study, the novel application of *in vivo* allograft 2D COSY spectroscopy revealed a 50% reduction in peak intensity from threonine, taurine, choline and creatine in individuals with advanced allograft dysfunction. Notably, taurine concentration in our patients was significantly altered only at the kidney graft level. Recent studies suggest that during kidney injury, transcriptional repression of the taurine transporter by p53 determines intracellular depletion of taurine, causing necrotic cell death [49]. On the other hand, taurine supplementation protects mesangial and tubular cells from high glucose or hypoxia *in vitro*, ameliorates nephrotic syndrome or diabetic nephropathy *in vivo* in animal models [31], and provides better outcomes in patients transplanted with kidneys from donors submitted to taurine preconditioning [50]. T3 patients also displayed intra-graft reduction in choline, a pivotal factor for the synthesis of cell membranes and cell-signaling components, a condition that can translate to acute renal failure and hypertension in animal models [51]. Finally, *in vivo* 2D COSY spectroscopy of the renal allograft revealed a reduction in creatine content, whose major function is the transport of high energy groups from mitochondria to cytoplasm, and is produced/stored in the kidney cortex; however, the implications of lack of creatine in kidney pathology are not clear yet. Notably, although not statistically different, a general increase in intra-graft lipid content among T3 patients was evident.

The limitations of our study include minor overlap of metabolomic profile of the imaging study with the targeted metabolomics of the biofluids, rendering it impossible for us to evaluate whether serum or urinary metabolites reflected systemic or local changes within the transplanted kidney, therefore, metabolomic disturbances identified here, will need to be studied further using tools of the functional studies. We also acknowledge a relatively small sample size as well as the cross-sectional nature of our study design. Finally, the patients included in our analysis were heterogenous in terms of both immunosuppressive schemes and other treatments that they were submitted to: the decision to opt for heterogeneous groups was based on the assumption of generalizability of our analysis. irrespective of underlying metabolomic alterations induced by exogenous treatments, while focusing on common patterns of metabolic abnormalities merely determined by the extent of kidney function. However, potential value of the candidate metabolites in predicting worsening kidney graft function will need to be evaluated in the subsequent follow-up studies.

We report the existence of a relationship between different levels of kidney graft impairment and imbalance of specific metabolites possibly linked to the pathophysiology of renal graft dysfunction. Low GFR was significantly associated with serum circulating factors linked to negative immunomodulation, hypertension, micro-ischemic events, fibrosis and cytotoxicity. Metabolic alterations at the parenchymal level of the transplanted kidney were also evident with significant reduction in high-energy and structural components of the graft parenchyma, and finally analysis of the urinary matrix highlighted the existence of a pro-hypertensive and pro-inflammatory environment within the transplanted organ.

## Supporting Information

S1 Data (DOCX)

S1 Table Overview of the total number of metabolites analyzed per biofluid (i.e. serum and urine) before and after threshold selection based on detection in at least 80% of the study patients (commonly detected). Metabolite concentrations are expressed as μM.
(DOCX)

S2 Table Numerical report of amino acids significantly different among groups in urine based on their alteration in serum. Data expressed as median (25th, 75th percentiles). Metabolite concentrations are expressed as μM.
(DOCX)

S3 Table No differences were detected among groups in branched chain amino acid (isoleucine, leucine, valine) concentration in serum and urine. Data expressed as median (25th, 75th percentiles). Metabolite concentrations are expressed as μM.
(DOCX)

S4 Table 2D Correlated Spectroscopy Crosspeak Assignments. (DOCX)

S1 Fig Representative 2D COSY voxel location as shown on 3 plane T2-weighted Magnetic Resonance Imaging. (DOCX)

## Abbreviations

1D-NMR: one dimensional-nuclear magnetic resonance
2D-COSY: two dimensional correlated spectroscopy
ADMA: asymmetric dimethylarginine
ATP: adenosine tri-phosphate
AU: arbitrary unit
C4: butyrylcarnitine
C12: Dodecenoylcarnitine
Ctrl: control
FIA-MS/MS: Flow Injection Analysis-mass spectrometry
GFR: glomerular filtration rate
H: hydrogen
HLA: human leukocyte antigen
IDO: indoleamine 2,3-dioxygenase
K: potassium
LC-MS/MS: liquid chromatography-mass spectrometry
Na: sodium
SDMA: symmetric dimethylarginine
T: tertile
TGF-β1: trasforming growth factor-β1
