# Supplementary material for: Metabolomic Profiling in Individuals with a Failing Kidney Allograft
Source: PLoS One. 2017 Jan 4;12(1):e0169077. doi: 10.1371/journal.pone.0169077 (PMC5214547; doi:10.1371/journal.pone.0169077)
Supplement: S1 Data — (DOCX) [file pone.0169077.s001.docx]

**Online Supplemental Appendix**

**Materials and methods**

**Selection of individuals**

The main causes of failure of the native kidneys among kidney transplant individuals were: unknown cause (n=9), chronic glomerulonephritis (n=15), diabetes mellitus (n=10), polycystic kidney disease (n=4) and nephronophthisis (n=2). Renal allograft individuals were on the following immunosuppressive therapeutic protocols: tacrolimus (Tac; n=5); CsA (n=1); Tac, mycophenolate mofetil (MMF; n=4); CsA and prednisone (n=3), Tac+prednisone (n=3), rapamycin and prednisone (n=2); Tac+MMF+prednisone (n=15), CsA+MMF (n=1), CsA+azathioprine (n=2), Tac+azathioprine (n=2), Tac+rapamycin (n=2).

**Kidney allograft function**

At the time of examination, individuals underwent renal function evaluation according to serum creatinine, albumin excretion rate (AER), glomerular filtration rate (GFR) and blood urea nitrogen content. Early morning spot urine samples were obtained for measurement of AER and were analyzed by immunonephelometry with N albumin kits (Behring, Somerville, NY). Urine creatinine was measured by the Jaffe creatinine reagent system (Roche). Glomerular filtration rate was estimated using the 4-variable Modification of Diet in Renal Disease Study equation [1] as follows: GFR (mL/min/1.73 m^2^ body surface area) = 186.3 × (serum creatinine)^−1.154^ × age^−0.203^ × 0.742 (if female). Laboratory measurements were performed centrally.

**Urine and blood sample collection**

In the *ex vivo* LC-MS/MS and FIA-MS/MS allograft individuals cohort, blood was drawn into dedicated serum tubes (BD EDTA Vacutainer®, Franklin Lakes, NJ) which were gently inverted twice and allowed to rest at room temperature for at least 30 min to obtain complete coagulation. For serum collection, blood was centrifuged at 15 ^◦^C for 10 min and then aliquoted into synthetic tubes, followed by storage at -80 ^◦^C until metabolomic analysis was conducted. Similarly, urine was collected into sterile urine containers (BD) and then transferred to synthetic tubes, which were subsequently stored at -80 ^◦^C. Samples were collected at the time of last routine follow-up clinical visit, corresponding to row “Follow-up (months)” (Table 1).

**Metabolomics protocol**

**LC-MS/MS and FIA-MS/MS.** A mass spectrometric-based metabolomic approach (AbsoluteIDQ^TM^ p180 kit assay, BIOCRATES Life Sciences AG, Innsbruck, Austria) was undertaken to determine the concentration of several metabolite classes in urine and serum samples including amino acids, biogenic amines, acylcarnitines, lysophosphatidylcolines, phosphatidylcolines and sphingomyelins and was used according to manufacturer's instructions. Metabolite concentrations of each sample were determined in a single analysis. Briefly, 10ul of sample was loaded onto the kit’s filter paper substrate and dried under nitrogen followed by addition of 20 uL of a 5% PITC (phenyl-isothiocyanate) solution for derivatization. Filter spots were submitted to nitrogen evaporator drying process. Methanol and 5 mM ammonium acetate were used for metabolite extraction. Metabolite analysis was carried out by FIA-MS/MS for acylcarnitines, lipids, and hexose and LC-MS/MS for amino acids and biogenic amines, using the AB SCIEX 4000 QTrap^TM^ mass spectrometer (AB SCIEX, Darmstadt, Germany) with electrospray ionization. Standard flow injection technique was applied for all measurements (two 20 μL injections for positive and negative ion detection mode) and MRM detection was employed for quantification. AB SCIEX 4000 QTRAP^TM^ mass spectrometer was controlled using Analyst 1.4.2 Software (AB SCIEX). The assay workflow was controlled using the Met*IDQ* Software (Biocrates Life Sciences AG), for sample registration, calculation of metabolite concentrations and export of data into additional data analysis software. Particularly, metabolite~~s~~ concentration detection and computation was performed by relating peak heights of analytes and chosen internal standards, using the, which contains built-in settings for validation of all metabolites. Concentrations of all analyzed metabolites were corrected for natural isotope distribution using algorithms developed by BIOCRATES and implemented in the Met*IDQ* software suite. Metabolite concentrations are reported in µM units. Urinary metabolite concentration values were normalized to creatinine according to the probabilistic quotient normalization in order to account for the impaired renal function in our cohorts. Data are expressed as median and interquartile range.

**Two dimensional correlated spectroscopy (2D COSY).** Localizer imaging sequences using non-breath held T2 weighted MR imaging in the three planes were acquired to locate the transplanted kidney with axial 3D-MPRAGE and reconstructed in the sagittal and coronal planes with 2 mm slice resolution for accurate localization of the voxel. 2D COSY was acquired in the transplanted kidney using a voxel size of 3x3x1.5 cm^3^ (13.5 cm^3^) located primarily in the cortex as shown in Figure S1 using Siemens Verio (Siemens AG, Erlangen, Germany) and the operating software VB17 (Siemens AG). Special care was taken to avoid contamination of abdominal and perirenal fat. COSY acquisition included a starting echo time of 30 ms, followed by 55 t1 increments with increment size of 0.8 ms. giving an indirect spectral width of 1250 Hz, TR 1.5 s, RF carrier frequency at 2.0 ppm, weak water suppression using WET, spectral width of 2000 Hz, and 6 averages per increment, with acquisition of 1024 data points. Due to minimal motion of the transplanted kidney, the 2D COSY data was not acquired with breath-holds or motion correction. Internal water reference was acquired using TE=135ms, TR=2000ms, 16 averages, no water suppression The total examination time was approximately 30 minutes.

2D COSY data were post-processed using custom software for 3D visualization as well as quantitatively using Felix-2007 (Accelrys, San Diego, CA), where crosspeak volumes were measured including location (F2, F1 in ppm) derived from previous publications [2,3], amplitude, and volume as shown in Table S4. Additional 3D reconstruction of the 2D COSY data was completed using custom MATLAB scripts that allowed for improved visualization of the crosspeaks similar to methods used in previous brain studies [4,5]. Water reference data were processed using LCmodel to obtain a water reference signal. All peak and crosspeak volumes were then normalized to the unsuppressed water signal [6] to compare between subjects.

**Results**

**Metabolite selection**

142 metabolites were commonly detected in serum, while 58 were identified in urine samples (S1 Table), and the most obvious differences between groups were found in amino acid, biogenic amine, and acylcarnitine levels in both biofluids.

**Low urinary glutamine and tryptophan confirm altered kidney graft amino acid uptake and systemic inflammation in patients with worse graft function**

Urinary glutamine concentration was overall lower in transplant individuals as compared to healthy non-allograft controls, a finding in agreement with the hypothesis of reduced renal uptake of amino acids [7], which then results in high glutamine levels in the serum of T3 individuals with impaired graft function (Figs 1: A and B). Specifically, glutamine was significantly reduced in T1 kidney transplant patients as compared to healthy individuals (T1=20 [12, 27] vs. Ctrl=40 [21, 44] μM, p=0.049; S2 Table). Similarly, the progressive reduction in urinary tryptophan following the loss of GFR in renal transplant patients suggests that the catabolism of this immunomodulating amino acid takes place at a systemic level (test for trend [T1-T3], p=0.05), (S2 Table).

**Serum and urinary branched-chain amino acids are not altered in kidney transplant individuals**

Finally, in our cohort, branched-chain amino acids (BCAA) were not different among groups of patients in serum and urine, in contrast to previous evidence demonstrating a link between impaired kidney function and BCAA serum levels [8], (S3 Table).

**References**

1. Levey AS, Bosch JP, Lewis JB, Greene T, Rogers N, et al. (1999) A more accurate method to estimate glomerular filtration rate from serum creatinine: a new prediction equation. Modification of Diet in Renal Disease Study Group. Ann Intern Med 130: 461-470.

2. Ramadan S, Andronesi OC, Stanwell P, Lin AP, Sorensen AG, et al. (2011) Use of in vivo two-dimensional MR spectroscopy to compare the biochemistry of the human brain to that of glioblastoma. Radiology 259: 540-549.

3. Ramadan S, Baltzer PA, Lin A, Stanwell P, Box H, et al. (2012) L-COSY of breast cancer at 3T. Eur J Radiol 81 Suppl 1: S129-131.

4. Lin AP, Ramadan S, Stern RA, Box HC, Nowinski CJ, et al. (2015) Changes in the neurochemistry of athletes with repetitive brain trauma: preliminary results using localized correlated spectroscopy. Alzheimers Res Ther 7: 13.

5. Mountford C, Quadrelli S, Lin A, Ramadan S (2015) Six fucose-alpha(1-2) sugars and alpha-fucose assigned in the human brain using in vivo two-dimensional MRS. NMR Biomed 28: 291-296.

6. Hammer S, de Vries AP, de Heer P, Bizino MB, Wolterbeek R, et al. (2013) Metabolic imaging of human kidney triglyceride content: reproducibility of proton magnetic resonance spectroscopy. PLoS One 8: e62209.

7. Fadel FI, Elshamaa MF, Essam RG, Elghoroury EA, El-Saeed GS, et al. (2014) Some amino acids levels: glutamine,glutamate, and homocysteine, in plasma of children with chronic kidney disease. Int J Biomed Sci 10: 36-42.

8. Cano NJ, Fouque D, Leverve XM (2006) Application of branched-chain amino acids in human pathological states: renal failure. J Nutr 136: 299S-307S.

**Supplementary tables and figures**

**S1 Table.** Overview of the total number of metabolites analyzed per biofluid (i.e. serum and urine) before and after threshold selection based on detection in at least 80% of the study patients (commonly detected). Metabolite concentrations are expressed as μM.

|  | **SERUM** | | | **URINE** | | |
| --- | --- | --- | --- | --- | --- | --- |
|  | Total (n) | Commonly detected (n) | % | Total (n) | Commonly detected (n) | % |
| Amino Acids (μM) | 42 | 31 | 73.8 | 42 | 37 | 88.1 |
| Acylcarnitines (μM) | 41 | 15 | 36.6 | 41 | 19 | 46.3 |
| Hexose (μM) | 1 | 1 | 100.0 | 1 | 1 | 100.0 |
| Phosphatidylcolines (μM) | 77 | 69 | 89.6 | 77 | 1 | 1.3 |
| Sphingomyelins (μM) | 15 | 14 | 93.3 | 15 | 0 | 0.0 |
| Lysophosphatidylcholines (μM) | 14 | 12 | 85.7 | 14 | 0 | 0.0 |
| *Total* | 190 | **142** | 74.7 | 190 | **58** | 30.5 |

**S2 Table.** Numerical report of amino acids significantly different among groups in urine based on their alteration in serum. Data expressed as median (25th, 75th percentiles). Metabolite concentrations are expressed as μM.

|  | | |  |  | ***T1-T3*** | ***T1 vs. Ctrl*** |
| --- | --- | --- | --- | --- | --- | --- |
|  | Ctrl | T1 | T2 | T3 | Test for Trend | p-value |
| Glutamine (μM) | 40 (21, 44) | 20 (12, 27) | 15 (8, 19) | 17 (14, 19) | ns | 0.049 |
| Tryptophan (μM) | 4.93 (3.27, 5.94) | 4.63 (4.35, 5.18) | 3.54 (2.24, 4.93) | 2.95 (2.04, 3.59) | 0.05 | ns |

**S3 Table.** No differences were detected among groups in branched chain amino acid (isoleucine, leucine, valine) concentration in serum and urine. Data expressed as median (25th, 75th percentiles). Metabolite concentrations are expressed as μM.

|  |  |  |  |  | ***T1-T3*** | ***T1 vs. Ctrl*** |
| --- | --- | --- | --- | --- | --- | --- |
| **SERUM** | Ctrl | T1 | T2 | T3 | Test for Trend | p-value |
| Isoleucine (μM) | 74 (63, 118) | 90 (74, 98) | 92 (79, 122) | 84 (63, 118) | ns | ns |
| Leucine (μM) | 131 (108, 195) | 165 (144, 199) | 169 (147, 199) | 158 (152, 186) | ns | ns |
| Valine (μM) | 235 (170, 282) | 254 (216, 290) | 267 (224, 329) | 264 (254, 294) | ns | ns |
| **URINE** |  |  |  |  |  |  |
| Isoleucine (μM) | 1.06 (0.82, 1.23) | 0.92 (0.80, 0.96) | 0.97 (0.74, 1.44) | 1.03 (0.81, 1.23) | ns | ns |
| Leucine (μM) | 2.98 (2.56, 3.50) | 3.64 (2.82, 7.72) | 2.61 (2.13, 3.45) | 2.66 (2.19, 3.99) | ns | ns |
| Valine (μM) | 2.83 (1.92, 2.94) | 2.09 (1.34, 2.37) | 1.91 (1.50, 2.39) | 1.83 (1.54, 2.39) | ns | ns |

**S4 Table**. 2D Correlated Spectroscopy Crosspeak Assignments.

| **Peak #** | **Name** | **F2** | **F1** |
| --- | --- | --- | --- |
| 1 | Water 1 |  |  |
| 2 | Olefinic Lipid | 5.51 | 5.54 |
| 3 | Water 2 | 4.72 | 4.72 |
| 4 | Homocarnosine | 4.46 | 4.49 |
| 5 | G' | 4.23 | 4.25 |
| 6 | Serine | 3.95 | 3.97 |
| 7 | Alanine/Glu | 3.73 | 3.72 |
| 8 | Threonine | 3.57 | 3.58 |
| 9 | Taurine | 3.44 | 3.43 |
| 10 | Choline 1 | 3.30 | 3.31 |
| 11 | Choline 2 | 3.10 | 3.10 |
| 12 | Creatine | 2.87 | 2.86 |
| 13 | Aspartate | 2.70 | 2.70 |
| 14 | Glutathione | 2.47 | 2.47 |
| 15 | Glutamate | 2.09 | 2.07 |
| 16 | Methylene | 1.85 | 1.85 |
| 17 | Methyl/Alanine | 1.23 | 1.23 |
| 18 | Lipid3 | 0.81 | 0.81 |
| 19 | E | 1.90 | 1.22 |
| 20 | Lipid4 | 1.22 | 0.68 |
| 21 | A | 0.87 | 1.28 |
| 22 | E' | 1.44 | 2.12 |
| 23 | D | 2.67 | 5.27 |
| 24 | C | 1.99 | 5.27 |
| 25 | G | 4.00 | 5.17 |

**Abbreviations.**

- **F1, F2**: frequency co-ordinates that localize the position of each peak in the 2D-COSY spectrum
- **Peak 5, G’**: Glycerol backbone R–(CO)–O–CH′H″–CH–O–(CO)–R
- **Peak 18, Lipid3**: Methyl endgroup of triglycerides –(CH_2_)*_n_*–CH_2_–CH_3_
- **Peak 19, E**: Methylene coupling –(CH_2_)*_n_*–CH_2_–CH_3_
- **Peak 20, Lipid 4**: Methyl-methylene coupling –(CH_2_)*_n_*–CH_2_–CH_3_
- **Peak 21, A**: Methyl-methylene coupling –(CH_2_)*_n_*–CH_2_–CH_3_
- **Peak 22, E’**: Methylene coupling –(CH_2_)*_n_*–CH_2_–CH_3_
- **Peak 23, D**: Mono-unsaturated fatty acids –CH=CH–CH_2_–CH=CH
- **Peak 24, C**: Poly-unsaturated fatty acids –CH=CH–CH_2_–CH=CH
- **Peak 25, G**: Glycerol backbone R–(CO)–O–CH′H″–CH–O–(CO)–R

**S1 Fig**. Representative 2D COSY voxel location as shown on 3 plane T2-weighted Magnetic Resonance Imaging.

**
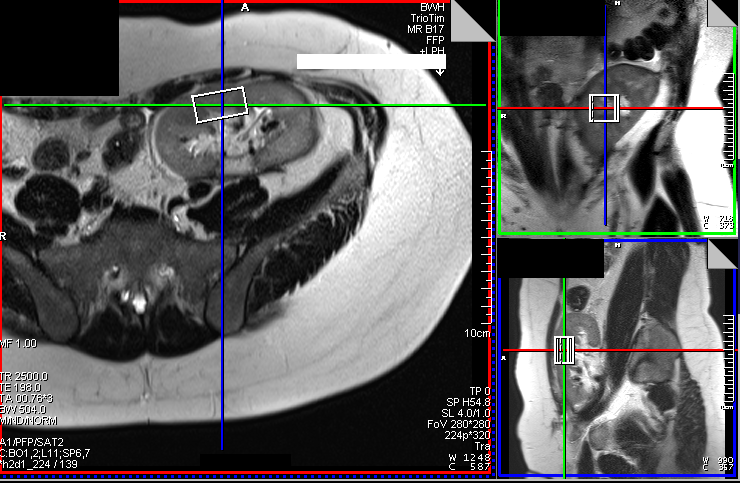
**
