# Supplementary material for: Metabolomic Profiling in Individuals with a Failing Kidney Allograft
Source: PLoS One. 2017 Jan 4;12(1):e0169077. doi: 10.1371/journal.pone.0169077 (PMC5214547; doi:10.1371/journal.pone.0169077)
Supplement: S1 Table — Metabolite concentrations are expressed as μM. (DOCX) [file pone.0169077.s002.docx]

**S1 Table.** Overview of the total number of metabolites analyzed per biofluid (i.e. serum and urine) before and after threshold selection based on detection in at least 80% of the study patients (commonly detected). Metabolite concentrations are expressed as μM.

|  | **SERUM** | | | **URINE** | | |
| --- | --- | --- | --- | --- | --- | --- |
|  | Total (n) | Commonly detected (n) | % | Total (n) | Commonly detected (n) | % |
| Amino Acids (μM) | 42 | 31 | 73.8 | 42 | 37 | 88.1 |
| Acylcarnitines (μM) | 41 | 15 | 36.6 | 41 | 19 | 46.3 |
| Hexose (μM) | 1 | 1 | 100.0 | 1 | 1 | 100.0 |
| Phosphatidylcolines (μM) | 77 | 69 | 89.6 | 77 | 1 | 1.3 |
| Sphingomyelins (μM) | 15 | 14 | 93.3 | 15 | 0 | 0.0 |
| Lysophosphatidylcholines (μM) | 14 | 12 | 85.7 | 14 | 0 | 0.0 |
| *Total* | 190 | **142** | 74.7 | 190 | **58** | 30.5 |
