# Supplementary material for: Metabolomic Profiling in Individuals with a Failing Kidney Allograft
Source: PLoS One. 2017 Jan 4;12(1):e0169077. doi: 10.1371/journal.pone.0169077 (PMC5214547; doi:10.1371/journal.pone.0169077)
Supplement: S2 Table — Data expressed as median (25th, 75th percentiles). Metabolite concentrations are expressed as μM. (DOCX) [file pone.0169077.s003.docx]

**S2 Table.** Numerical report of amino acids significantly different among groups in urine based on their alteration in serum. Data expressed as median (25th, 75th percentiles). Metabolite concentrations are expressed as μM.

|  | | |  |  | ***T1-T3*** | ***T1 vs. Ctrl*** |
| --- | --- | --- | --- | --- | --- | --- |
|  | Ctrl | T1 | T2 | T3 | Test for Trend | p-value |
| Glutamine (μM) | 40 (21, 44) | 20 (12, 27) | 15 (8, 19) | 17 (14, 19) | ns | 0.049 |
| Tryptophan (μM) | 4.93 (3.27, 5.94) | 4.63 (4.35, 5.18) | 3.54 (2.24, 4.93) | 2.95 (2.04, 3.59) | 0.05 | ns |
