# Supplementary material for: Metabolomic Profiling in Individuals with a Failing Kidney Allograft
Source: PLoS One. 2017 Jan 4;12(1):e0169077. doi: 10.1371/journal.pone.0169077 (PMC5214547; doi:10.1371/journal.pone.0169077)
Supplement: S3 Table — Data expressed as median (25th, 75th percentiles). Metabolite concentrations are expressed as μM. (DOCX) [file pone.0169077.s004.docx]

**S3 Table.** No differences were detected among groups in branched chain amino acid (isoleucine, leucine, valine) concentration in serum and urine. Data expressed as median (25th, 75th percentiles). Metabolite concentrations are expressed as μM.

|  |  |  |  |  | ***T1-T3*** | ***T1 vs. Ctrl*** |
| --- | --- | --- | --- | --- | --- | --- |
| **SERUM** | Ctrl | T1 | T2 | T3 | Test for Trend | p-value |
| Isoleucine (μM) | 74 (63, 118) | 90 (74, 98) | 92 (79, 122) | 84 (63, 118) | ns | ns |
| Leucine (μM) | 131 (108, 195) | 165 (144, 199) | 169 (147, 199) | 158 (152, 186) | ns | ns |
| Valine (μM) | 235 (170, 282) | 254 (216, 290) | 267 (224, 329) | 264 (254, 294) | ns | ns |
| **URINE** |  |  |  |  |  |  |
| Isoleucine (μM) | 1.06 (0.82, 1.23) | 0.92 (0.80, 0.96) | 0.97 (0.74, 1.44) | 1.03 (0.81, 1.23) | ns | ns |
| Leucine (μM) | 2.98 (2.56, 3.50) | 3.64 (2.82, 7.72) | 2.61 (2.13, 3.45) | 2.66 (2.19, 3.99) | ns | ns |
| Valine (μM) | 2.83 (1.92, 2.94) | 2.09 (1.34, 2.37) | 1.91 (1.50, 2.39) | 1.83 (1.54, 2.39) | ns | ns |
