# Supplementary material for: Metabolomic Profiling in Individuals with a Failing Kidney Allograft
Source: PLoS One. 2017 Jan 4;12(1):e0169077. doi: 10.1371/journal.pone.0169077 (PMC5214547; doi:10.1371/journal.pone.0169077)
Supplement: S4 Table — (DOCX) [file pone.0169077.s005.docx]

**S4 Table**. 2D Correlated Spectroscopy Crosspeak Assignments.

| **Peak #** | **Name** | **F2** | **F1** |
| --- | --- | --- | --- |
| 1 | Water 1 |  |  |
| 2 | Olefinic Lipid | 5.51 | 5.54 |
| 3 | Water 2 | 4.72 | 4.72 |
| 4 | Homocarnosine | 4.46 | 4.49 |
| 5 | G' | 4.23 | 4.25 |
| 6 | Serine | 3.95 | 3.97 |
| 7 | Alanine/Glu | 3.73 | 3.72 |
| 8 | Threonine | 3.57 | 3.58 |
| 9 | Taurine | 3.44 | 3.43 |
| 10 | Choline 1 | 3.30 | 3.31 |
| 11 | Choline 2 | 3.10 | 3.10 |
| 12 | Creatine | 2.87 | 2.86 |
| 13 | Aspartate | 2.70 | 2.70 |
| 14 | Glutathione | 2.47 | 2.47 |
| 15 | Glutamate | 2.09 | 2.07 |
| 16 | Methylene | 1.85 | 1.85 |
| 17 | Methyl/Alanine | 1.23 | 1.23 |
| 18 | Lipid3 | 0.81 | 0.81 |
| 19 | E | 1.90 | 1.22 |
| 20 | Lipid4 | 1.22 | 0.68 |
| 21 | A | 0.87 | 1.28 |
| 22 | E' | 1.44 | 2.12 |
| 23 | D | 2.67 | 5.27 |
| 24 | C | 1.99 | 5.27 |
| 25 | G | 4.00 | 5.17 |

**Abbreviations.**

- **F1, F2**: frequency co-ordinates that localize the position of each peak in the 2D-COSY spectrum
- **Peak 5, G’**: Glycerol backbone R–(CO)–O–CH′H″–CH–O–(CO)–R
- **Peak 18, Lipid3**: Methyl endgroup of triglycerides –(CH_2_)*_n_*–CH_2_–CH_3_
- **Peak 19, E**: Methylene coupling –(CH_2_)*_n_*–CH_2_–CH_3_
- **Peak 20, Lipid 4**: Methyl-methylene coupling –(CH_2_)*_n_*–CH_2_–CH_3_
- **Peak 21, A**: Methyl-methylene coupling –(CH_2_)*_n_*–CH_2_–CH_3_
- **Peak 22, E’**: Methylene coupling –(CH_2_)*_n_*–CH_2_–CH_3_
- **Peak 23, D**: Mono-unsaturated fatty acids –CH=CH–CH_2_–CH=CH
- **Peak 24, C**: Poly-unsaturated fatty acids –CH=CH–CH_2_–CH=CH
- **Peak 25, G**: Glycerol backbone R–(CO)–O–CH′H″–CH–O–(CO)–R
