# Supplementary material for: Metabolomic Profiling in Individuals with a Failing Kidney Allograft
Source: PLoS One. 2017 Jan 4;12(1):e0169077. doi: 10.1371/journal.pone.0169077 (PMC5214547; doi:10.1371/journal.pone.0169077)
Supplement: S1 Fig — (DOCX) [file pone.0169077.s006.docx]

**S1 Fig**. Representative 2D COSY voxel location as shown on 3 plane T2-weighted Magnetic Resonance Imaging.

**
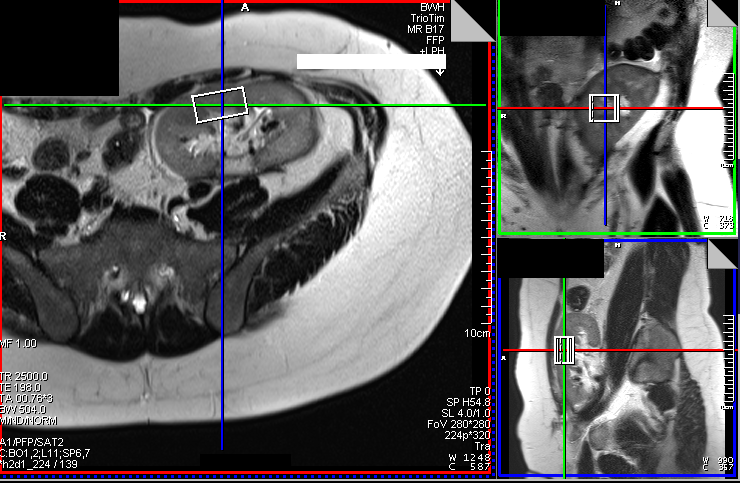
**
